# “Dance Like Nobody’s Watching”: Exploring the Role of Dance-Based Interventions in Perceived Well-Being and Bodily Awareness in People With Parkinson’s

**DOI:** 10.3389/fpsyg.2020.531567

**Published:** 2020-11-05

**Authors:** Rebecca Hadley, Olivia Eastwood-Gray, Meryl Kiddier, Dawn Rose, Sonia Ponzo

**Affiliations:** ^1^Department of Psychology and Sports Sciences, University of Hertfordshire, Hatfield, United Kingdom; ^2^Ready Steady Move, Chelmsford, United Kingdom; ^3^School of Music, Lucerne University of Applied Sciences and Arts, Lucerne, Switzerland

**Keywords:** Parkinson’s disease, bodily awareness, dance, body appreciation, well-being, exercise

## Abstract

Evidence indicates that bodily perception is negatively related to Parkinson’s disease (PD); in particular, people with Parkinson’s (PwP) feel dissatisfaction in their physical abilities and appearance. While established treatments exist to ameliorate motor symptoms in PD, research has yet to explore the effects of well-being-focused interventions in relation to the subjective experience of bodily concerns of PwP. This mixed methods exploratory study investigated the constructs of body appreciation in relation to well-being in PwP and the impact of participation in a dance class on body appreciation and well-being, comparing PwP with age-matched controls. Participants (*n* = 27 PwP, *n* = 14 controls) completed the Warwick Edinburgh Mental Well-Being Scale and the Body Appreciation Scale before and after taking part in a dance class. Well-being was positively associated with body appreciation in PwP (*r*_*s*_ = 0.64, *p* < 0.001) but not in controls. Following participation in a dance class, all participants’ well-being scores increased; a greater increase in well-being scores was observed for controls. A pilot qualitative study explored bodily awareness with PwP who attended dance classes (*n* = 4) and other movement-based activities (*n* = 4). Analysis of the interview data indicated that PwP who danced showed heightened bodily awareness, including bodily limitations, in comparison with PwP who did not dance. These preliminary findings provide initial insight explaining the lack of improvements in body appreciation in PwP following a dance class. The current study highlights the need for dance interventions for PwP to consider incorporating elements that encourage a body positive attitude alongside fostering perceived well-being.

## Introduction

Parkinson’s disease (PD) is a chronic neurodegenerative condition with an approximated worldwide prevalence rate of 425 per 100,000 persons aged 65–74 years (Pringsheim et al., 2014). The number of individuals living with the condition globally is estimated to have doubled between 1990 and 2016 to over 6 million (Dorsey et al., 2018). Clinical presentation of motor symptoms typically includes slowness of movement, muscular rigidity, and/or tremor (Kalia and Lang, 2015). Non-motor difficulties also present in PD include neuropsychiatric conditions (e.g., anxiety and depression), sleep disorders, fatigue, and pain (Chaudhuri et al., 2011). Even in the early stages, PwP report that non-motor symptoms have a greater impact on health-related quality of life than motor features (Pfeiffer, 2016). As the severity of the condition progresses, worsening of both motor and non-motor symptoms may affect an individual’s ability to undertake essential daily tasks, such as washing and dressing (Duncan et al., 2014). The complex interplay between motor and non-motor symptoms leads to reduced physical activity (Nimwegen et al., 2011) and multifaceted stressors on psychological well-being (Nicoletti et al., 2017) for PwP.

The majority of studies that have focused on non-motor symptoms (e.g., depression; Okun and Watts, 2002, general well-being; Jenkinson et al., 1995) has not considered the relationship between PD and bodily perception as a mediating factor (Koller, 1984). Existing evidence indicates a relationship between PD and bodily perception (Koller, 1984) as well as dissatisfaction toward one’s own body’s physical abilities and appearance (Koller, 1984**; Gamarra et al., 2009**). Accordingly, neuroimaging findings (see Christopher et al., 2014 for a meta-analysis) suggest that bodily concerns in PD may be linked to damage to the insular cortex, a key region involved in bodily awareness (namely, awareness of one’s own body; Craig, 2002). Despite growing evidence, little is known about the impact of bodily related concerns on PwP quality of life. Only a few studies (e.g., Ghielen et al., 2017) propose integrated approaches to increase PwP well-being while taking into account bodily awareness.

In other conditions, including fibromyalgia, chronic fatigue syndrome (Courtois et al., 2015), post-traumatic stress disorder (Mehling et al., 2018), and anorexia nervosa (Kolnes, 2017), exercise has been used as a therapeutic strategy that can enhance bodily awareness and improve well-being. Dance, in particular, is associated with a heightened connectedness to the body as dancers communicate through performing physical movement (Walter and Yanko, 2018). For example, contemporary dance has been associated with higher body appreciation (i.e., satisfaction with and acceptance of one’s own body appearances and abilities; Avalos et al., 2005; Tylka and Wood-Barcalow, 2015) in dancers compared to non-dancers (Swami and Tovée, 2009). Furthermore, taking part in dance has been found to improve self-reported positive well-being in young adults (Kim and Kim, 2007) and reduce depressive symptoms in older adults living in a nursing home (Vankova et al., 2014). For PwP, dance classes have been recognized as being useful in the management of both the physical and psychological components of well-being (Reynolds et al., 2016). Specifically, dance has been shown to increase the well-being of attendees due to its high level of engagement and unique social components (Shanahan et al., 2015). Quantitative studies have demonstrated significant meaningful improvement in quality of life for PwP after regular participation in tango dance classes (Hackney and Earhart, 2009). An increase in psychological well-being has also been reported for PwP after attending a single dance session (Koch et al., 2016). Qualitative research has indicated that the social value of dancing with a partner, or as a group, fosters relationships and reduces feelings of loneliness (Houston and McGill, 2013; Holmes and Hackney, 2017). However, few studies have considered how dancing affects body appreciation and whether this construct may be related to perceived well-being in PwP. A more comprehensive assessment of such mediating factors, i.e., bodily awareness and appreciation, may help health professionals understand more about potential barriers to participation and inform their practice.

Previous studies have suggested that dance, as a body-focused activity, may benefit perceived well-being in PwP, although the role that bodily awareness and appreciation might play has not been studied in relation to PwP. Therefore, the present study explored the relationship between perceived well-being and body appreciation, as well as the effect of dance classes on both constructs in PwP and controls. Specifically, the aims of the current study were to investigate quantitatively (a) whether the constructs of well-being and body appreciation were correlated with one another in PwP compared to controls and (b) whether taking part in a dance class shapes well-being and body appreciation and specifically so in PwP compared with age-matched controls. We predicted (a) that self-reported well-being would be positively associated with body appreciation for PwP and for controls and (b) higher body appreciation and well-being for both PwP and age-matched controls post-dance class compared to pre-scores. Semi-structured interviews were undertaken as part of a pilot qualitative study to gain further insight into how bodily awareness is experienced by PwP who attend dance classes and PwP who attend other types of movement-based activities, excluding dance.

## Quantitative Study

### Methods

#### Participants

A total of 41 people, 27 with Parkinson’s (16 female, age range = 47–86 years, *M* = 69.04, *SD* = 8.56 months, average age at diagnosis = 63 years) and 14 controls (10 female, age range = 52–81 years, *M* = 68.57, *SD* = 8.72), were recruited via dance classes specifically devised for PwP throughout Hertfordshire and the surrounding counties. Controls (*n* = 14) were the partners or caregivers of the PwP and attended the same classes. Exclusion criteria was a diagnosis of a neurological disorder other than Parkinson’s but did not include psychiatric disorders due to the comorbidity with PD (e.g., depression; Schapira et al., 2017). The majority of participants (PwP and controls) were physically active each week (Supplementary Table 1) and reported dance as one of the activities they participated in (PwP; *n* = 23 and controls; *n* = 12). Most participants with PD (*n* = 26) were taking dopaminergic medication. This study was approved by the Health, Sciences, Engineering and Technology Ethics Committee with Delegated Authority at the University of Hertfordshire [Protocol reference: aLMS/UG/UH/03538(3) and aLMS UG UH 03539(1)]. All participants gave informed, written consent.

#### Materials

Two dependent variables were collected; well-being was assessed using the Warwick–Edinburgh Mental Well-Being Scale (WEMWBS; Tennant et al., 2007) and positive body image assessed using the Body Appreciation Scale-2 (BAS-2; Tylka and Wood-Barcalow, 2015).

The WEMWBS is a self-report measure capturing the hedonistic and eudemonic elements of positive mental well-being (Tennant et al., 2007). It includes 14 items such as, “I’ve been feeling useful” and “I’ve been feeling good about myself.” A 5-point Likert scale (1 = none of the time and 5 = all of the time) is provided for each item. The minimum score is 14, and the maximum is 70. The overall score is calculated by summing the scores across all items. A higher score indicates higher mental well-being. The WEMWBS has been found to have a high internal reliability for a general population sample (Cronbach’s α = 0.91; Tennant et al., 2007).

The BAS-2 is a self-report measure of an individual’s acceptance and positive perceptions of their body. It includes 10 items such as, “I respect my body.” and “I am attentive to my body’s needs.” Each item is rated on a 5-point scale (1 = never and 5 = always). The minimum score is 10, and the maximum score is 50. The total score is calculated by summing the scores across all items. A higher score indicates greater body appreciation. The BAS-2 has been found to have a high internal reliability for a general population sample (Cronbach’s α = 0.97; Tylka and Wood-Barcalow, 2015).

#### Experimental Procedure

Demographic information collected included symptoms of Parkinson’s, current medications, and types of physical activity regularly undertaken.

Participants (control and PwP) completed the WEMWBS and BAS-2 (both in written form) immediately before (pre) and after (post) taking part in a singular “Dance for Parkinson’s” class. For the few PwP who had difficulty completing the handwritten forms, either their partner or a volunteer helped record their responses.

Each dance class was an hour long and began with a 10–15 min seated warm-up. To accommodate a range of Parkinson’s severities, the sessions (30–40 min) included dance steps that could be performed to music while seated or standing. Unlike other studies that have used a specific dance style (i.e., Tango Argentino; Hackney and Earhart, 2009; Koch et al., 2016; Beerenbrock et al., 2020), the current study used a genre classified as social dance (Lewis et al., 2016). This required participants to perform solo and partnered routines of a variety of styles (i.e., Bollywood, Tango, Irish dance). The last 10 min of the class involved a seated cool-down.

#### Data Analysis

Non-parametric statistical tests were employed for data analysis due to the small sample size of the control group and the non-normal distribution of WEMWBS and BAS-2 scales (see Figures 3, 4 below). To test whether wellbeing and body appreciation improved for PwP following participation in a dance class (compared with controls), main effects of (i) group (PwP vs Controls) and (ii) time (pre vs post) were calculated. The main effect of group was calculated by averaging the pre and post WEMWBS and BAS-2 scores for the PD and control group separately. Values were then compared between groups using a Mann-Whitney test. The main effect of time was calculated by averaging the values of pre and post WEMWBS and BAS-2 scores, regardless of group classification. These values were compared via a Wilcoxon signed-rank test. To assess the presence of two-way interactions between group and time, post-dance-values of WEMWBS and BAS-2 were subtracted from pre-dance values and differences between the two groups analyzed using Mann–Whitney *U*-tests. *Post hoc* tests (Bonferroni corrected α = 0.025) were employed to investigate significant two-way interactions (tested via Wilcoxon signed-rank tests). Effect sizes were computed using Cohen’s $r(r=Z/\sqrt{N)}$. Spearman correlations between WEMWBS and BAS-2 baseline (pre) scores in (i) the Parkinson’s sample and (ii) the controls were conducted to assess the relationship between the two constructs irrespective of time.

One control participant (female, aged 75 years) did not complete the WEMWBS, and one control (female, aged 69 years) and one PwP (male, aged 72 years) did not complete the BAS-2. These data were not included in the analyses.

Data were analyzed and plotted using R (Allen et al., 2019).

### Results

#### Correlations

To investigate whether the constructs of well-being and body appreciation are related, only the pre-intervention scores were used, and their relationship was explored via Spearman correlations (two-tailed) in the Parkinson’s sample as well as in controls. Pre-intervention data, unaffected by the experimental manipulation, were used, as they reflect the stable state of these traits.

**FIGURE 1:**
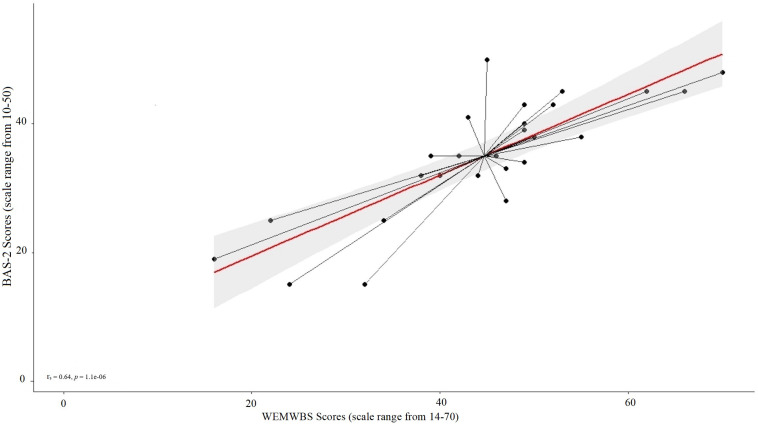
Scatterplot of the mean values for the Parkinson’s sample of pre-intervention ratings on the wellbeing (WEMWBS; scale ranges from 14–70; a higher score indicates higher wellbeing) and the body appreciation scale (scale ranges from 10–50; a higher score indicates higher appreciation).

**FIGURE 2:**
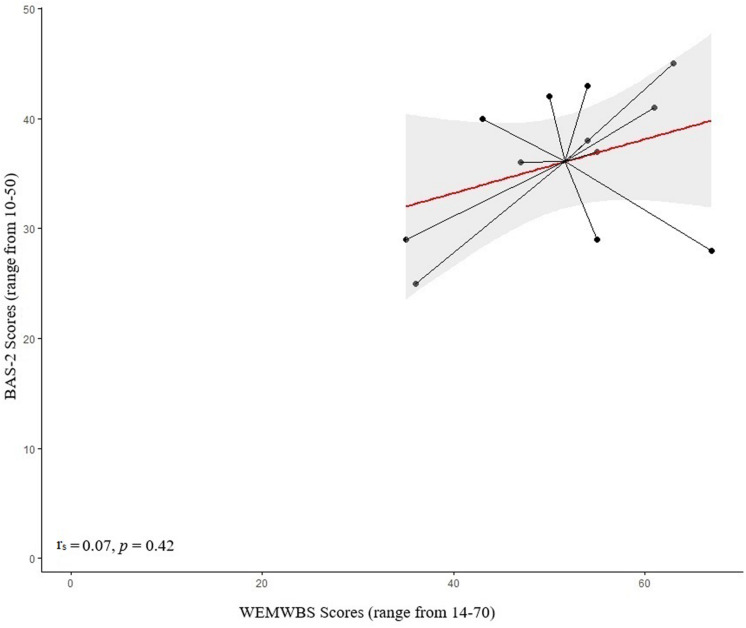
Scatterplot of the mean values for the control sample of pre-intervention ratings on the wellbeing (WEMWBS; scale ranges from 14–70; a higher score indicates higher wellbeing) and the body appreciation scale (scale ranges from 10–50; a higher score indicates higher appreciation).

A significant positive correlation was found between well-being scores (as measured via the WEMWBS) and body appreciation scores (BAS-2) in the Parkinson’s sample [*r*_*s*_(24) = 0.64, *p* < 0.001; Figure 1]. However, no significant correlation was found between well-being and body appreciation scores in the control sample [*r*_*s*_(10) = 0.07, *p* = 0.42; Figure 2]. This finding suggests that, for PwP specifically, increases in body appreciation are associated with increases in perceived well-being, but this is not the case for controls. However, the lack of effect in the control group may be due to the insufficient experimental power, given the small sample size.

#### Well-Being

To investigate whether participation in a dance class shapes perceived well-being (measured using the WEMWBS) in PwP compared with age-matched controls, Mann-Whitney U-tests were used to analyze between-subjects factor of a 2 (groups: PwP vs. control) × 2 (time: pre vs. post) mixed design. Within-subjects factors were analyzed via Wilcoxon signed-rank tests. A significant main effect of time overall (*Z* = -4.02, *p* < 0.001, *r* = 0.64) revealed that post-dance scores were higher than pre-dance ones (pre: *median* = 47.50, *interquartile range* = 12.50, *M* = 46.83, *SD* = 11.78; post: *median* = 51.50, *interquartile range* = 18.00, *M* = 51.05, *SD* = 12.45).

**FIGURE 3:**
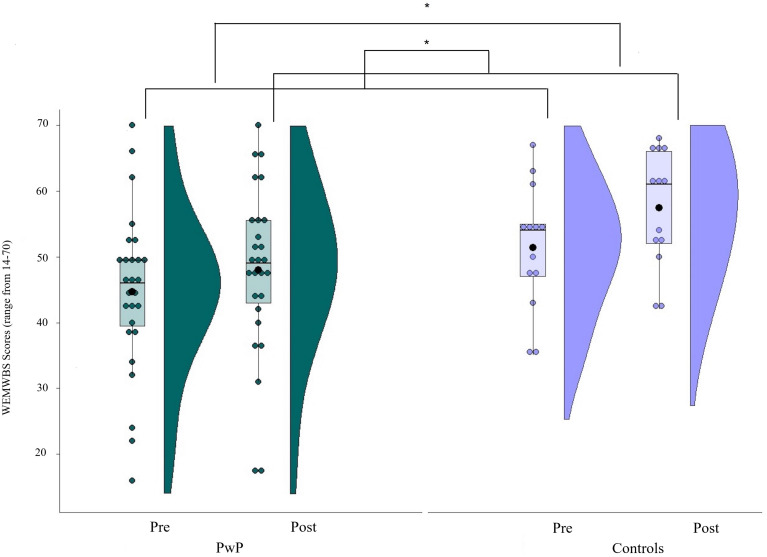
Mean values of the wellbeing (WEMWBS). Scores on a scale from 14 (lower wellbeing) to 70 (higher wellbeing); Solid line = median; Black dot = mean; Whiskers: upper whisker = min(max(x), Q_3 + 1.5*IQR); lower whisker = max(min(x), Q_1–1.5*IQR.

A main effect of group was found on well-being scores (*Z* = -1.96, *p* = 0.05, *r* = 0.31), with controls showing higher levels of well-being in comparison with PwP (PwP: *median* = 48.00, *interquartile range* = 9.75, *M* = 46.3, *SD* = 12.29; controls: *median* = 55.00, *interquartile range* = 14.50, *M* = 54.42, *SD* = 8.81; Figure 3).

However, the interaction between group and time was not significant (*Z* = -1.46, *p* = 0.14, *r* = 0.23).

#### Body Appreciation

**FIGURE 4:**
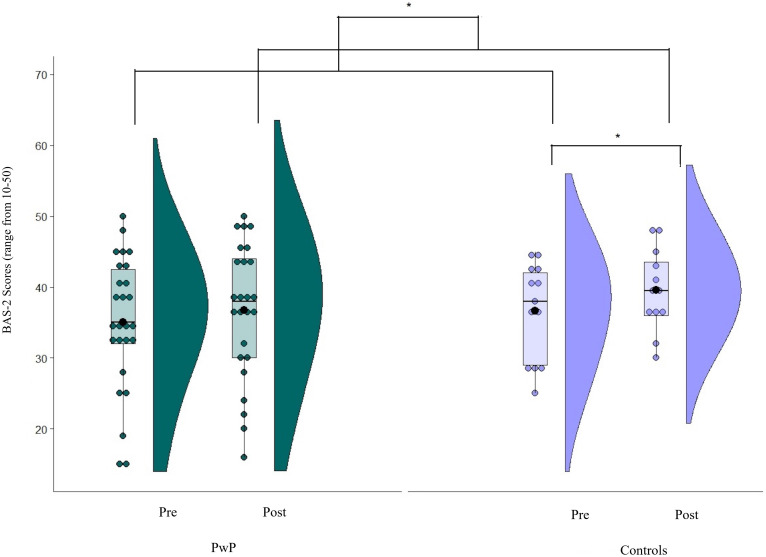
Mean values of the body appreciation (BAS-2). Scores on a scale from 10 (lower body appreciation) to 50 (greater body appreciation); Solid line = median; Black dot = mean; Whiskers: upper whisker = min(max(x), Q_3 + 1.5*IQR); lower whisker = max(min(x), Q_1–1.5*IQR.

To investigate whether participation in a dance class shapes body appreciation (measured using the BAS-2) in PwP compared with age-matched controls, Mann-Whitney U-tests were used to analyze between-subjects factor of a 2 (groups: PwP vs. control) × 2 (time: pre vs. post) mixed design. Within-subjects factors were analyzed via Wilcoxon signed-rank tests. A significant main effect of time (*Z* = -2.62, *p* = 0.01 *r* = 0.42) was found, with post-dance scores higher than pre-dance ones (pre: *median* = 37.00, *interquartile range* = 12.00, *M* = 35.56, *SD* = 8.63; post: *median* = 38.00, *interquartile range* = 12.50, *M* = 38.1, *SD* = 11.55; Figure 4). No main effect of group was found (*Z* = -0.94, *p* = 0.35, *r* = 0.15).

A significant two-way interaction was revealed between group and time (*Z* = -2.05, *p* = 0.04, *r* = 0.33). Further investigation via Bonferroni-corrected *post hoc* tests indicated that the control group’s body appreciation scores increased more following dance intervention than PwP (controls: *Z* = -2.72, *p* = 0.007, *r* = 0.53; pre: *median* = 38.00, *interquartile range* = 13.00, *M* = 36.69, *SD* = 6.79; post: *median* = 40.00, *interquartile range* = 9.00, *M* = 42.69, *SD* = 12.5; PwP: *Z* = -1.07, *p* = 0.28, *r* = 0.21; pre: *median* = 35.00, *interquartile range* = 10.50, *M* = 35, *SD* = 9.5; post: *median* = 37.50, *interquartile range* = 14.00, *M* = 35.81, *SD* = 10.55).

## Qualitative study

### Methods

#### Participants

Participants were recruited from a dance class at the University of Hertfordshire, local activity groups, and via word of mouth by those who attended the groups. To be eligible for the study, participants were required to have a diagnosis of Parkinson’s and no other diagnosed movement or neurological condition. Initially, 10 PwP were recruited for the interviews, although only 8 were finally able to participate. All participants were taking dopaminergic medication, and none reported any changes to their medication in the weeks prior to participating in the study.

The participants included four individuals who regularly attended dance classes (range = 4–7 years) at the University of Hertfordshire (three female, age range = 46–70, *M* = 60.75, *SD* = 12.42). Two PwP had participated in the quantitative part of the study. The severity of Parkinson’s ranged from mild to severe (Hoehn and Yahr stage 1–4) and years living with the condition ranged from 5 to 17. In addition to dance, the participants took part in a range of other activities, such as seated yoga, cycling, and an aerobic exercise group. The reported time spent being physically active ranged from 1–2 h to 3–4 h per week.

The remaining four participants (three male, age range = 50–78, *M* = 67.75, *SD* = 12.42) did not participate in the dance classes but engaged in other types of physical activity including golf, leisure walking, and aqua aerobics. The severity of Parkinson’s ranged from mild to moderate (Hoehn and Yahr stage 1–3) and years living with the condition ranged from 2 to 14. The reported time spent being physically active ranged from 2–3 h to more than 4 h per week. This study was approved by an institutional Ethics Committee and all participants gave informed, written consent.

#### Interview Structure

Semi-structured interview schedules (see Supplementary Material) were developed based on reviewed research and sought to explore how participants felt about their bodies both physically and psychologically in the past and at present. Feelings toward the body may include thoughts about physical functionality, attitudes (Clancy, 2010), and appearance (Pila et al., 2016). The semi-structured approach was chosen because it allows for open-ended questions to gather data grounded in the experience of the participant while also incorporating questions guided by constructs of interest to the researchers (Galletta, 2013).

#### Interview Setting

Individual interviews were conducted with PwP who regularly participated in Dance for Parkinson’s classes at the University of Hertfordshire and with PwP who exercised but did not dance. All interviews were conducted by a psychology student at the university campus. Two of the participants who did not dance requested that their partners be present during the interview; however, neither partner contributed to the interview. Each interview lasted approximately 30–45 min and was audiorecorded using an Olympus digital voice recorder.

#### Analysis

In order to maintain the full richness of the data and conversational nature of the interview, naturalized transcription was used (Oliver et al., 2005). In line with Braun and Clarke’s (2006) analysis recommendations in the instance of an *a priori* research focus (the effect of dance on bodily awareness in the current study), the authors used theoretical thematic analysis to analyze the transcripts. The transcripts were subsequently coded independently by two psychologists (RH and DR) according to *a priori* hierarchical themes. This allows for the further development of previous research insights pertinent to the topic (Gamarra et al., 2009; Ghielen et al., 2017; Beerenbrock et al., 2020). The five *a priori* codes were (1) dance and the body (Houston and McGill, 2013; Soundy et al., 2014; Koch et al., 2016; Beerenbrock et al., 2020), (2) body functionality, (3) body frustration, (4) pre/post-diagnosis changes in bodily awareness (Taleporos and McCabe, 2002), and (5) body dissatisfaction (Gamarra et al., 2009). Where utterances supported *a priori* codes, they were classified accordingly. Where text offered novel insight, new labels and descriptions were assigned. These were later discussed by the two researchers until agreement was reached regarding the nature of their contribution to the extant literature. In order to contextualize the utterance, participants who attended a dance class were titled “PD-dancer” (PwP who danced). Participants who did not dance were titled “PD-no-dance” (PwP who did not dance but took part in other movement-based activities).

### Findings

The title and description of the *a priori* codes were reviewed and retitled, and subthemes were added to reflect the data in this study (Supplementary Table 2). Thematic mind maps were then used to explore the relationships between the *a priori* and novel codes (see Supplementary Figures 6, 7). The respective findings are described in order of contribution to the following research questions: (1) How do participants perceive their body in relation to their condition in general? (2) How is awareness of the body experienced by participants who dance and those who take part in other movement-based activities? (3) Which aspects of dancing are related to participants perceptions of their body? Selected themes and subthemes that elaborate on the quantitative findings of the current study and add insight to the results of the reviewed literature are considered below (full results in Supplementary Materials).

Theme 1 Parkinson’s and body perception

In relation to PwP general perspectives about their condition and its impact on their ability to engage in the activities they would like to do, two subthemes were identified (physical limitations and Parkinson’s symptoms). First, the struggles and frustration both groups of participants felt with their own bodies due to their condition were evident, as illustrated in the following comments:

“Like getting dressed I get frustrated. You think I must be able to find the armholes”

(PD4-Dancer)

*“It makes me tired. It makes me frustrated because I can’t do things as well as I used to*”

(PD1-Dancer)

“If it wasn’t for the Parkinson’s I’d be doing all sorts of things.” (PD5-No-dance)

The second subtheme (Parkinson’s symptoms) captures the uncertainty associated with symptom variability and the perception of lack of control being problematic, as the following examples convey:

“I guess the frustrating thing about Parkinson’s is that you can do something one minute and then half an hour later you can’t do it, and there’s no explanation why you can’t do it some days” (PD3-Dancer)

“I get very annoyed with my limbs at times. When I wash my hair in this kind of motion this hand often just stops, and I’m doing this and I’m thinking well, you know – do something!”

(PD7-No-dance)

However, some participants expressed being able to move past the frustration to a place of acceptance even with the unpredictable nature of their symptoms:

“I can accept that I have them [symptoms of Parkinson’s] because I control them – because I have some agency over them. Whereas in the past, it was more difficult to accept and as they felt like they were beginning to control me” (PD7-No-dance).

Outside of movement-based activities, navigating everyday activities with Parkinson’s highlighted how other peoples’ lack of understanding regarding Parkinson’s were at odds with participants own perception of their body’s functionality.

Theme 2 Perceptions of the functionality of their own body

The second theme compares the impact of different perspectives and types of activities on body perception in relation to Parkinson’s. Three contributing factors were found: (a) independence, (b) comparing to others, and (c) other people’s understanding of PD.

The issue of independence observes the way in which both no-dance and dance participants perceived their body as an important factor in maintaining their autonomy.

Regardless of whether they danced or not, it was notable that participants described what their body enabled them to do rather than focusing on aesthetics, as the following extract demonstrates:

“I can still ride a two-wheel bicycle. So, my balance isn’t too bad.” (PD4-Dancer)

Participant PD7-No-dance listed several activities that they were able to do without needing help and expressed gratitude for the independence a functioning body gave them:

“I can walk around people and I can go through doorways without freezing…I can dance and I can [DJ] mix. I can make food. I am fine on self-care.”

“I am grateful that I have a body that transports me where I want to go under my own steam.”

The subtheme of comparing to others captures participants’ perceptions of their body as an important factor in challenging their own view of Parkinson’s. Comparisons between themselves and other PwP who attended the same activities were also a way to monitor their status; a litmus test for the progression of their own symptoms as the following excerpts demonstrate:

“*When you are with other people you realize their problems are greater than yours or otherwise if I think that’s probably one guy that’s better off than me. I mean, he’s been diagnosed 3 years I’ve been diagnosed five so that’s the difference, but you’ve got to take in what’s going on around you.” (PD8-No-dance)*

“Being involved with other people that have Parkinson’s who are far worse than I am makes me realize how lucky I am at the moment to be just at this stage after 6 years.”(PD1-Dancer)

Theme 3 The impact of a dance class on bodily awareness

This theme captures PwP perceptions of dance classes as a space for bringing awareness to their ability to participate in the class (subtheme: negative awareness) and in which their preconceptions of Parkinson’s were challenged (subtheme: comparison to others with PD).

In the dance class, comparisons with able-bodied student volunteers were experienced in a positive way as this exemplar shows:

“*The mix of able-bodied people and disabled people together is good and I quite like it when people like you [the researcher] can’t do things.*” *(PD4-Dancer)*

Participating in dance drew attention in a negative way to changes in movement quality, as indicated below:

“*It reminds me that I’m getting old and I can’t move as freely as I did.” (PD1-Dancer)*

For some participants, this awareness meant coming to terms with uncontrollable changes associated with Parkinson’s, as the following quote highlights:

“*I think if you do exercise you think that’s going to fix me but then you realize that the fact that you can’t do it isn’t because you’re not fit enough…[but] because it’s coming from your brain.*” (*PD3-Dancer*)

Participants’ uncertainty of their own future with Parkinson’s contributed to an initial apprehension about attending a dance class:

“I would say that it was the only thing that really worried me before I came, that I was going to see a path mapped out in front of me.” (PD2-Dancer)

However, the same participant described a sense of enablement from seeing others with PD successfully take part in the dance classes despite initial worries that they would experience the opposite effect:

“People that have been diagnosed and living with it way longer than me, are still getting up and dancing. In fact, it’s amazingly encouraging, and it keeps you going really.”

(PD2-Dancer)

Motivation was also gained from the opportunity to track the progression of their condition against other PwP at the class:

“*It’s a kind of indicator of where you were at, where you are now, and kind of keep going*.” *(PD4-Dancer)*

Overall, the social aspect of attending specialized dance classes were described as very positive experiences.

## Discussion

The current study explored the relationship between perceived well-being and body appreciation, as well as the effect of dance classes on both constructs in PwP and age-matched controls. We found that perceived well-being and body appreciation (pre-intervention) were significantly positively correlated in PwP but not in the control sample. Previous studies have shown a positive relationship between body appreciation and specific dimensions of perceived well-being in healthy individuals (e.g., self-esteem, Avalos et al., 2005; Swami et al., 2009 and self-compassion, Homan and Tylka, 2015; Marta-Simões and Ferreira, 2019). However, in the current study, the association between these constructs was specific to PwP rather than controls. This finding should be interpreted with caution given the small size of the control sample, and further research is needed to shed light on these conflicting results. To the best of our knowledge, no study to date has investigated the relationship between well-being and body appreciation in the context of PwP. Further research is needed to examine the potential clinical implications of body-focused interventions, such as dance, for PwP, taking into consideration the relationship between well-being and body appreciation.

The second aim of the current study was to investigate whether participation in a dance class would lead to improvements in perceived well-being and body appreciation. Findings suggest that taking part in dance classes improved well-being for PwP and controls. Previous studies have shown an effect of dance on mood (Lewis et al., 2016) and quality of life (Heiberger et al., 2011; Westheimer et al., 2015; Aguiar et al., 2016), even following a brief intervention (4 weeks; Blandy et al., 2015; singular workshop; Koch et al., 2016). The current findings confirm that dance, even as a singular session, may be a useful therapeutic intervention that is psychologically beneficial for all attendees. However, the improvement in well-being post-dance class was slightly more pronounced for controls compared to PwP. Few studies include a control group that undertake the same dance intervention as PwP when investigating the effect of dance on well-being. Therefore, future studies should explore the extent to which dance interventions may benefit perceived well-being for PwP relative to people without PD, such as the partners and caregivers of PwP.

Previous research with healthy individuals has shown regular dance participation to be associated with higher body appreciation (ballet and contemporary dance; Swami and Harris, 2012, street dance; Swami and Tovée, 2009) and enhanced body image (Burgess et al., 2006). In line with these studies, we found a greater increase in body appreciation scores for the age-matched control group following participation in a dance class, supporting the suggestion that dance may foster body enhancement in non-experts (Langdon and Petracca, 2010). However, no specific improvement was found in body appreciation for PwP. To the best of our knowledge, there are no other studies to date that have investigated the impact that dance interventions may have on perceived body appreciation for PwP. The lack of findings on body appreciation as reported in the current study may be due to a heightened bodily awareness following dance classes. It may be speculated that such an increase in bodily awareness may not necessarily translate into appreciation but may lead to insights into PwP’s physical limitations. Preliminary qualitative findings from the current study suggest that participants who danced experienced bodily awareness as a change in movement quality and an understanding that Parkinson’s imposes limitations on their ability to participate in the class. Future studies could incorporate body compassion practices into interventions with PwP to help them transition from bodily awareness to body appreciation.

Qualitative findings of the present study suggest that participating in dance classes may draw negative awareness to the body in PwP, particularly in terms of functional capabilities and limitations, and that PwP tended to critically compare themselves with others. Therefore, dance interventions for PwP aiming to foster improvements in perceived well-being may benefit from incorporating elements that encourage body appreciation. Increased bodily awareness, including movement deficits and remaining mobility, have recently been reported by Beerenbrock et al. (2020) who explored the impact of tango classes on the body experience of PwP. Beerenbrock et al. (2020) suggest that body-related concerns are likely to be compounded by symptoms of the condition, such as impairments in balance and gait, which can lead to lower self-confidence in one’s ability to carry out activities of daily living and a fear of falling (Palakurthi and Burugupally, 2019). The lack of control that PwP perceive to have over their body, as described by both the dance and no-dance participants in the qualitative study, alongside higher body-related concerns, may result in PwP becoming detached from their bodies (Beerenbrock et al., 2020). Hence, dance may lead to heightened bodily awareness, which may, in turn, negatively impact body appreciation following dance interventions. Future studies should investigate the extent to which increased bodily awareness may lead to higher or lower levels of body appreciation in PwP.

The findings of this current exploratory study should be considered in light of some contextual limitations. With regard to recruitment, it should be noted that some participants regularly attend dance classes, and this was not a novel experience, a factor that may have led to a positive bias toward dance. Due to the lack of systematic control over participants’ dance experience, it is not known whether the effect of dance on well-being and body appreciation can be generalized to novice dancers. The effect found in the current study may be limited to participants who had more experience of dancing. Future studies should consider controlling for participant experience with dancing. The results of the quantitative study highlight that participation in a singular hour-long dance class may result in immediate benefit in terms of well-being and body appreciation. However, from these results, it is not known whether this effect is consistent or remains over time. Future studies should include a long-term follow-up to address this. The quantitative study included a limited control group that took part in the same dance class as the PwP. The inclusion of another group undertaking a different type of exercise would shed light on whether changes in well-being and body appreciation are specific to dance or exercise more generally. Given that the control group predominantly included of PwP partners, it could be suggested that the well-being of controls was improved by proxy due to perceived improvement in the well-being of their partner with PD. While there is some supporting qualitative evidence for this argument (Prado et al., 2020), we believe that this would reflect an important finding in itself, as it indicates the value in attending dance for both groups. In particular, the findings of Prado et al. (2020) also indicated that caregivers may attend dance classes, as there is not enough time for them to do something else on their own. Therefore, joint interventions such as dance may provide an important opportunity that might not otherwise be afforded for some caregivers to benefit, psychologically, even if this is through the improved well-being of their partner. The control group was also not of equal size to the Parkinson’s group. The discrepancy in sample size between the Parkinson’s and control group is in line with several other studies that have investigated the physical and psychological benefits of dance for PwP (Romenets et al., 2015; Lewis et al., 2016) and reflects a genuine imbalance between those who attend classes with partners and those who do not (Prado et al., 2020). The quantitative study did not include a no-dance PD control group. Future studies could consider including people with greater disease progression and more severe symptoms in order to understand how the nature of this degenerative condition impacts on individuals’ bodily concerns in relation to dance. Finally, the qualitative study was limited to interviews with eight PwP; therefore, the results should be treated as preliminary. Future studies should consider including a larger sample size when exploring the effect of dance on bodily awareness in Parkinson’s.

Overall, a positive relationship between dancing and well-being was observed in this study. However, no effect of dance was found on body appreciation in PwP, and qualitative data suggested that the participants’ focus was on potential physical limitations, rather than body appreciation, which was heightened by the comparison with other classes’ attendees. Taken together, these findings suggest that dance interventions aiming to foster well-being in PwP should take into consideration the repercussions on perceived body appreciation, by adopting a more bodily positive attitude throughout the intervention and explicitly tackling its potential impact on PwP.

## Data Availability Statement

The datasets generated for this study are available on request to the corresponding author.

## Ethics Statement

The studies involving human participants were reviewed and approved by the Health, Sciences, Engineering and Technology Ethics Committee with Delegated Authority at the University of Hertfordshire. The participants provided their written informed consent to participate in this study.

## Author Contributions

OE-G conducted the interviews, transcribed the qualitative data, and contributed to the manuscript. RH and SP collected the quantitative data and supervised the study. Qualitative data analysis was undertaken by RH and DR and quantitative by SP and RH. MK aided the recruitment of participants from local dance classes and reviewed the manuscript. Revisions of the manuscript were undertaken by RH, SP, and DR. All authors contributed to the article and approved the submitted version.

## Conflict of Interest

MK leads the dance classes that take place at the University of Hertfordshire. The remaining authors declare that the research was conducted in the absence of any commercial or financial relationships that could be construed as a potential conflict of interest.
