# Supplementary material for: “Dance Like Nobody’s Watching”: Exploring the Role of Dance-Based Interventions in Perceived Well-Being and Bodily Awareness in People With Parkinson’s
Source: Front Psychol. 2020 Nov 5;11:531567. doi: 10.3389/fpsyg.2020.531567 (PMC7674205; doi:10.3389/fpsyg.2020.531567)
Supplement: Supplementary file 1 [file Data_Sheet_1.docx]

Supplementary material

**Supplementary table 1.** Absolute frequency (%) of hours spent being physically active per week.

| Hours | Group | |
| --- | --- | --- |
|  | Parkinson’s | Control |
| 1-2 | 9 (33) | 3 (21) |
| 2-3 | 6 (22) | 1 (7) |
| 3-4 | 4 (15) | 4 (29) |
| More than 4 | 8 (30) | 4 (29) |
| Missing | - | 2 (14) |
| Total | 27 (100) | 14 (100) |

**Supplementary table 2.** Frequencies of supporting utterances associated with *a priori* themes and related sub-themes.

| Evolution of *A priori* codes | Absolute Frequency (%) | Sub-themes |
| --- | --- | --- |
| 1. Components of dance that contribute to perceptions of the body (refined) | 73 (23) | 1. Comparison to others with PD 2. Normalisation 3. Positivity 4. Negative Awareness |
| 2. Contributing factors in the perception of body functionality (refined) | 55 (17) | 1. Independence 2. Comparing to others 3. Other people’s understanding of PD |
| 3. The role of Parkinson’s in feelings towards the body (refined | 54 (17) | 1. Physical abilities 2. Symptoms of Parkinson’s |
| 4. Changes in feelings towards one's own body pre and post PD diagnoses (no change) | 97 (31) | 1. Attitude 2. Desire to stay active |
| 5. Strategies to manage body dissatisfaction (refined) | 38 (12) | 1. Fluctuating attitude 2. Rejection of Parkinson’s identity |
| Total | 317 (100%) |  |

**Supplementary table 3**. Summary of fixed and random effects of linear mixed models for body appreciation (BAS-2).

|  | **BAS-2** | | |
| --- | --- | --- | --- |
| *Predictors* | *Estimates* | *CI* | *p* |
| (Intercept) | 37.69 | 24.23 – 51.15 | **<0.001** |
| BD analysis$pre post | -4.38 | -11.16 – 2.39 | 0.205 |
| BD analysis$Group | -3.50 | -13.02 – 6.02 | 0.471 |
| BD_analysis$pre_post:BD_analysis$Group | 5.19 | 0.40 – 9.98 | **0.034** |
| **Random Effects** | | | |
| σ^2^ | 25.89 | | |
| τ_00_ _ID_ | 74.97 | | |
| ICC | 0.74 | | |
| N _ID_ | 39 | | |
| Observations | 78 | | |
| Marginal R^2^ / Conditional R^2^ | 0.07/ 0.76 | | |

**Supplementary table 4**. Summary of fixed and random effects of linear mixed models for perceived wellbeing (WEMBS).

|  | **WEMBS** | | |
| --- | --- | --- | --- |
| *Predictors* | *Estimates* | *CI* | *p* |
| (Intercept) | 37.28 | 24.35 – 50.22 | **<0.001** |
| BD analysis$pre post | 0.59 | -4.43 – 5.61 | 0.818 |
| BD analysis$Group | 4.01 | -5.19 – 13.21 | 0.393 |
| BD_analysis$pre_post:BD_analysis$Group | 2.74 | -0.83 – 6.31 | 0.132 |
| **Random Effects** | | | |
| σ^2^ | 14.56 | | |
| τ_00_ _ID_ | 120.59 | | |
| ICC | 0.89 | | |
| N _ID_ | 40 | | |
| Observations | 80 | | |
| Marginal R^2^ / Conditional R^2^ | 0.13 / 0.91 | | |

Qualitative findings

**Theme 1.** *Parkinson’s and body perception.*

**Supplementary figure 1.** *Theme 1: Parkinson’s and body perception*

This first theme (see supplementary figure 1) captures the struggles and frustration people with Parkinson’s felt with their own bodies due to their condition, as illustrated in the following comments:

*“Like getting dressed I get frustrated. You think I must be able to find the armholes” (PD4-Dancer)*

*“It makes me tired. It makes me frustrated because I can't do things as well as I used to*” (*PD1-Dancer)*

*“if it wasn’t for the Parkinson’s I’d be doing all sorts of things.”* *(PD5-No-dance)*

A fine line between frustration and resentment appeared mediated by vulnerability as is apparent here:

“*People make allowances for me. I can see them doing it. It’s really annoying but it is also difficult because if I can't open the lid off something and somebody comes and does it for me then they're in trouble because you know, you've got to let me do it myself. I don't need you to do this for me. But if there's nobody there and I say will somebody come and do this for me, please, you know, I get stroppy because it's just frustration*” *(PD3- Dancer)*

The second sub-theme (Parkinson’s symptoms) captures the uncertainty associated with symptom variability and the perception of lack of control being problematic, as the following examples convey:

*“I guess the frustrating thing about Parkinson’s is that you can do something one minute and then half an hour later you can't do it, and there's no explanation why you can't do it some days”* *(PD3- Dancer)*

*“I get very annoyed with my limbs at times. Washing my hair is another good example. When I wash my hair in this kind of motion this hand often just stops, and I'm doing this and I'm thinking well, you know - do something! You know, get on with it! You should be able to operate and it's mostly the lack of dopamine. You get how it works, you get the mechanics, but it doesn't stop the frustration.”* *(PD7-No-dance)*

However, some participants expressed being able to move past the frustration to a place of acceptance even with the unpredictable nature of their symptoms:

*“I can accept that I have them [symptoms of Parkinson's] because I control them - because I have some agency over them. Whereas in the past, it was more difficult to accept and as they felt like they were beginning to control me”* *(PD7-No-dance).*

**Theme 2.** *PwP perceptions of the functionality of their own body*

**Supplementary figure 2.** *Theme 2: PwP perceptions of the functionality of their own body*

The second theme captures the way in which people with Parkinson’s perceived their body as an important factor in maintaining their autonomy (independence), and for challenging their own (comparing) and others’ perceptions (understanding) of Parkinson’s as illustrated in supplementary figure 2.

Regardless of whether or not they danced, it was notable that participants described what their body enabled them to do, rather than focusing on aesthetics, as the following extract demonstrates:

“*I can still ride a two-wheel bicycle. So, my balance isn't too bad” (PD4-Dancer)*

Participant PD7-No-Dance listed several activities they were able to do without needing help and expressed gratitude for the independence a functioning body gave them:

*“I am grateful that I have a body that transports me where I want to go under my own steam”( PD7-No-dance)*

Comparisons between themselves and other people with Parkinson’s was a way to monitor their status; a litmus test for the progression of their own symptoms as the following excerpts demonstrate:

“*When you are with other people you realize their problems are greater than yours or otherwise if I think that's probably one guy that's better off than me. I mean, he’s been diagnosed three years I've been diagnosed five so that's the difference, but you've got to take in what's going on around you” (PD8-No-dance)*

*“being involved with other people that have Parkinson's who are far worse than I am makes me realise how lucky I am at the moment to be just be at this stage after 6 years”(PD1-No-dance)*

In the dance class, comparisons with able-bodied student volunteers was experienced in a positive way as this exemplar shows:

“*the mix of able-bodied people and disabled people together is good and I quite like it when people like you [the researcher] can’t do things*” *(PD4- Dancer)*

However, several participants spoke at length about how other peoples’ lack of understanding regarding PD were at odds with their own perception of their body’s functionality, as PD3- Dancer describes:

“*I finally gave in and got parking badge and its really useful, but my problem is not that I can't I can't walk or anything. It's that I can't get out of the car if I can't open the door far enough and I'm getting close to other people. So, I think, well, I'm getting out of the car. I can walk, people are thinking what is she doing with a disabled badge and I feel like I'd have to explain it to them*.”

The same participant provided further insight in relation to how this experience had led to a change in behaviours so as to reduce the perceived inconvenience for others:

*“People don't understand why you can't do something. I know people get annoyed at me in the checkout. But now I've kind of changed the way I do things so that I don't make as much of an impact on anybody else. So, I try to you know, I'll go shopping twice rather than going once and get to it half as much each time.” (PD3-Dancer)*

**Theme 3.** *The impact of dance on bodily awareness*

**Supplementary figure 3.** *Theme 3: The impact of dance on bodily awareness*

The third theme captures PwP perceptions of dance classes to be a space for bringing awareness to their ability to participate in the class (sub-theme: negative awareness) and in which their preconceptions of Parkinson’s were challenged (sub-theme: comparison to others with PD), as illustrated in supplementary figure 3.

Participating in dance drew attention in a negative way to changes in movement quality, as indicated below:

“*It reminds me that I'm getting old and I can't move as freely as I did.” (PD1-Dancer)*

For some participants this awareness meant coming to terms with uncontrollable changes associated with Parkinson’s, as the following quote from PD3-Dancer highlights:

“*I think if you do exercise you think that's going to fix me and but then you realise that the fact that you can't do it isn't because you're not fit enough not because you haven't been doing exercise because it's coming from your brain*” (*PD3-Dancer*)

Participants uncertainty of their own future with Parkinson’s contributed to an initial apprehension about attending a dance class:

*“I would say that it was the only thing that really worried me before I came, that I was going to see a path mapped out in front of me” (PD2-Dancer)*

*“A couple of times I was quite shocked by what I saw. Some people are quite seriously afflicted with it [Parkinson’s], you know, you think that might be me down the line. I try not to think about it because you don't know what's in your own future really” (PD3-Dancer)*

However, several participants spoke at length about how their perceptions of people with Parkinson’s had changed once they had spent time with and got to know individuals at the class, as illustrated in the extracts below:

*“You learn that people are people and they are not a bag of symptoms and that everyone manages things is different ways. It’s quite inspirational and quite helpful to be amongst other people who have got similar things, my attitude has changed a lot” (PD2-Dancer)*

*“What I find in that class is that everyone is so sparky and with it and have led interesting lives and still are leading interesting lives.” (PD2-Dancer)*

*“It's such a varied disease just looking at the dance class there are so many different problems that people have, no one's got everything wrong” (PD3-Dancer)*

Dance classes provided a support network for participants, and were experienced as an empathetic activity rather than purely as exercise, as the following excerpts indicate:

*“Makes me feel normal. Being around other people with Parkinson's has a huge benefit. Yeah, absolutely because we help each other. We might not talk but the very fact that we're together. It's a help and it's a comfort.” (PD1-Dancer)*

*“It makes life easier because I understand what my fellow classmates are going through. We gee each other along it and keep going and support each other.” (PD1-Dancer)*

*“It definitely makes you feel better. It definitely sort of opens it up a bit instead of you feeling like you're trying to hide it [Parkinson’s] most of the time and just trying to be normal and mingling with the crowd” (PD3-Dancer)*

Several participants expressed a sense of energy and vibrancy gained from dance:

*“It makes me feel good about myself makes me makes me alive. Yeah, you've got a happy person.” (PD1-Dancer)*

*“I always feel happy after it, yeah, it makes me feel good and a bit more confident I think and also it does sort of free things up.” (PD2-Dancer)*

*“Immediately afterwards I always feel like my mood is good and uplifted” (PD2-Dancer)*

*“Dance is just fun. It's definitely makes you feel different.” (PD3-Dancer)*

Enablement of motivation stemmed from seeing other people with Parkinson’s overcome barriers to take part in the dance, as indicated in the following quotes from PD2-Dancer:

*“The reality of seeing people who sometimes really struggle to put one foot in front of the other but then it switches off for them and they dance, yeah that’s amazing. It’s the best present I could have been given really.”*

*“People that have been diagnosed and living with it way longer than me, are still getting up and dancing. In fact, it’s amazingly encouraging, and it keeps you going really.”*

For PD4-Dancer, motivation was also gained from the opportunity to track the progression of their condition against other people with Parkinson’s at the class:

“*It's a kind of indicator of where you were at, where you are now, and kind of keep going*.”

Overall, the social aspect of attending specialised dance classes were described as very positive experiences. The next theme provides some initial insights as to the ways in which PwP manage perceptions of their body.

Theme 4. *Strategies employed by PwP to manage body dissatisfaction*

**Supplementary figure 4.** *Theme 4: Strategies employed by PwP to manage body dissatisfaction*

The fourth theme (see supplementary figure 4) captures the way in which people with Parkinson’s experienced fluctuating attitudes regarding their body’s functionality. The theme also captured the feelings and moments in which people with Parkinson’s perceived their body dissatisfaction to be worsened, and the strategies adopted to manage such feelings. The “rejection of a Parkinson’s identity” predominantly reflects the experience of the participants who had not danced. Whereas, it is worth noting that utterances that provided novel insight regarding positive attitudes towards body appearance and functionality within this theme were exclusively made by those who danced.

Some participants were able to overcome their despair and adopt a more positive outlook, as the following quotes from PD1-Dancer illustrate:

*“I'm plodding along behind thinking why am I? Why am I here? What am I doing?”*

*“You have to accept it because you know, you've got it [Parkinson’s]. There's nothing you can do about it, except the best. So, that's what I do. I don't get up in the morning and think I've got Parkinson's. I've got to get on with it.”*

In addition to acceptance, maintaining a positive attitude was expressed as a sense of gratitude:

*“I'm still living, I’m slim, fit and I'm literally fit for my age. So, I’ve got stamina.”*

*(PD4-Dancer)*

*“I think that I’m really happy about my body. I'm in shape, you know.” (PD4-Dancer)*

However, the overwhelming experience of their Parkinson’s symptoms was challenging for some participants, as indicated in the extracts below:

*“when my Parkinson’s symptoms are really painful I kind of loose everything. I can’t speak, my brain goes a bit fuzzy, it has quite an impact. If my feet seize up it just sort of effects everything, not just my feet” (PD2-Dancer)*

*“I feel so bad that I don’t almost feel anything” (PD2-Dancer)*

These negative feelings seemed to be heightened during periods of loneliness:

“ *I think you can get depressed about things very easily with Parkinson's. It is depressing when you spend too much time on your own. You definitely get a bit miserable.”* *(PD3-Dancer)*

*“Yes, if I’m alone and it’s, you know, if the symptoms are being a nuisance its harder to get rid of the negative thoughts” (PD2-Dancer)*

*“It’s more difficult to be motivated when I’m alone. I think it’s easy to spiral and I think being alone is a condition of when that would happen”* *(PD2-Dancer)*

Feeling worse was also associated with breaks from physical activities, as illustrated in the following extract:

*“Last week nothing was on, like the other [Parkinson’s exercise] class I do on a Thursday wasn’t on and by the end of the week I felt drained because I hadn’t done anything” (PD2-Dancer)*

For the participants who did not attend dance classes, taking part in activities where they were likely to be confronted with the reality of how their symptoms might progress was a source of psychological discomfort, as illustrated by the quotes below:

*“We didn't really want to go along to the classes because I was worried about what states these people were going to be in” (PD5-No-dance)*

*“Being around people with worse symptoms of Parkinson's is a reminder of how things might be in the future. So, being around a lot of people that are showing similar symptoms can be a bit, I suppose it's a bit soul-destroying” (PD7-No-dance)*

Avoidance of activities with groups of other people with Parkinson’s helped participants manage this fear:

“*I don't mix that often with Parkinson’s patients*” *(PD5-No-dance)*

“*Do I want to be mixing with a lot of Parkinson's patients, you know, do I want that to be a bigger part of my life and probably right now probably not”* *(PD7-No-dance)*

For PD7-No-dance, distancing oneself from a Parkinson’s identity was part of a need for normalcy rather than being perceived and experienced as different, as demonstrated in the extract below:

*“I really really don't want to identify with, I don't want to identify myself or be identified as that Parkinson's person*.”

*“I'm not the Parkinson's patient that's not my big label you know. This is sort of this over-generalized label and I don't want to be, I don't want to fit. I certainly don't think of myself that way”*

**Theme 5:** *Changes in feelings towards one's own body pre and post PD diagnoses*

**Supplementary figure 5.** *Theme 5: Changes in feelings towards one's own body pre and post PD diagnoses*

Participants described how they felt about their bodies in relation to their diagnosis; attitudes were positive and negative (see supplementary figure 5). One participant described an overriding sense of injustice in receiving the diagnosis, followed by resignation, as illustrated below:

*“I'm angry about it. It seems unfair because you know life isn't fair, but I'm too young to have that. Yeah, I haven't done anything wrong. I'm not being an alcoholic. I haven't sniffed glue. I haven't done any of these things” (PD3-Dancer)*

*“Yeah, I thought life won't be the same again” (PD3-Dancer)*

In contrast, other people with Parkinson’s seemed to have adjusted well, or even found reason to become more accepting of their body;

*“I was very very frightened, extremely frightened. Yeah, and I guess a little bit depressed for a little while. But I realized that only I could make it better in myself. Yeah, so I thought let's just get on with it now”* *(PD1-Dancer)*

*“I'm constantly battling with my weight. But since Parkinson's, it doesn't seem that important. I’m kinder to my body since Parkinson's.” (PD1-Dancer)*

*“well, it doesn’t look better, but I would say I feel better about my body than before I was diagnosed” (PD2-Dancer)*

The theme also captured the way in which people with Parkinson’s perceived physical activity as a means of protest against the diagnosis while also supporting a positive outlook.

For PD5-No-dance, a sense of psychological defiance seemed to be closely tied to their ability to remain physically active, as indicated in the following quotes:

*“you have got to be accepting, either that or you will go under and I'm not one of these people to just sit at home and vegetate”* *(PD5-No-dance)*

*“I want to get out and not let the bastard get me down, so to speak” (PD5-No-dance)*

For several participants, engaging in physical activity facilitated feelings of accomplishment with what their body was able to achieve:

*“I know it’s hard to believe but I do marginally more exercise than I used to and that does make me feel better” (PD2-Dancer)*

*“I do a seated yoga class. I feel quite positive, like my body can do more things”*

*(PD2-Dancer)*

For PD5-No-dance this desire to stay active formed part of a positive doing cycle in which feeling better about their body’s capabilities resulted in motivation to undertake more activity:

*“I feel very pleased that I'm able to do the exercises better than I expected”*

*(PD5-No-dance*)

*“when I get home I want to do more exercise and I've got kettlebells at home and a cross trainer so I can get on those” (PD5-No-dance*)

For others staying active was important in alleviating both depression and feelings of loneliness, as seen in the following excerpt:

*“I just do as much exercise as I can and social stuff because it makes it a more positive experience really and it’s helped in the sense, that it [Parkinson’s] is a bit depressing” (PD3-Dancer)*


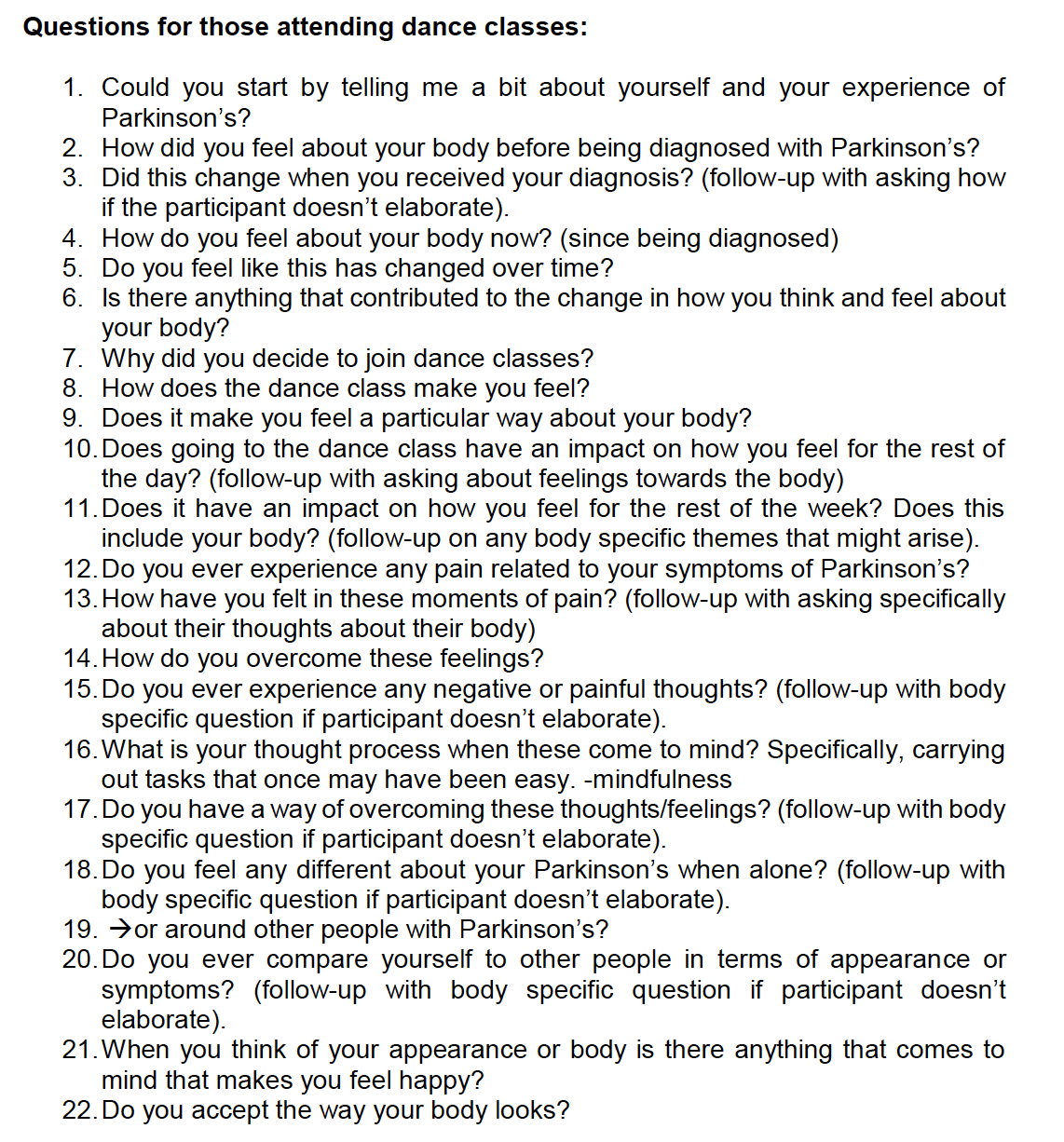


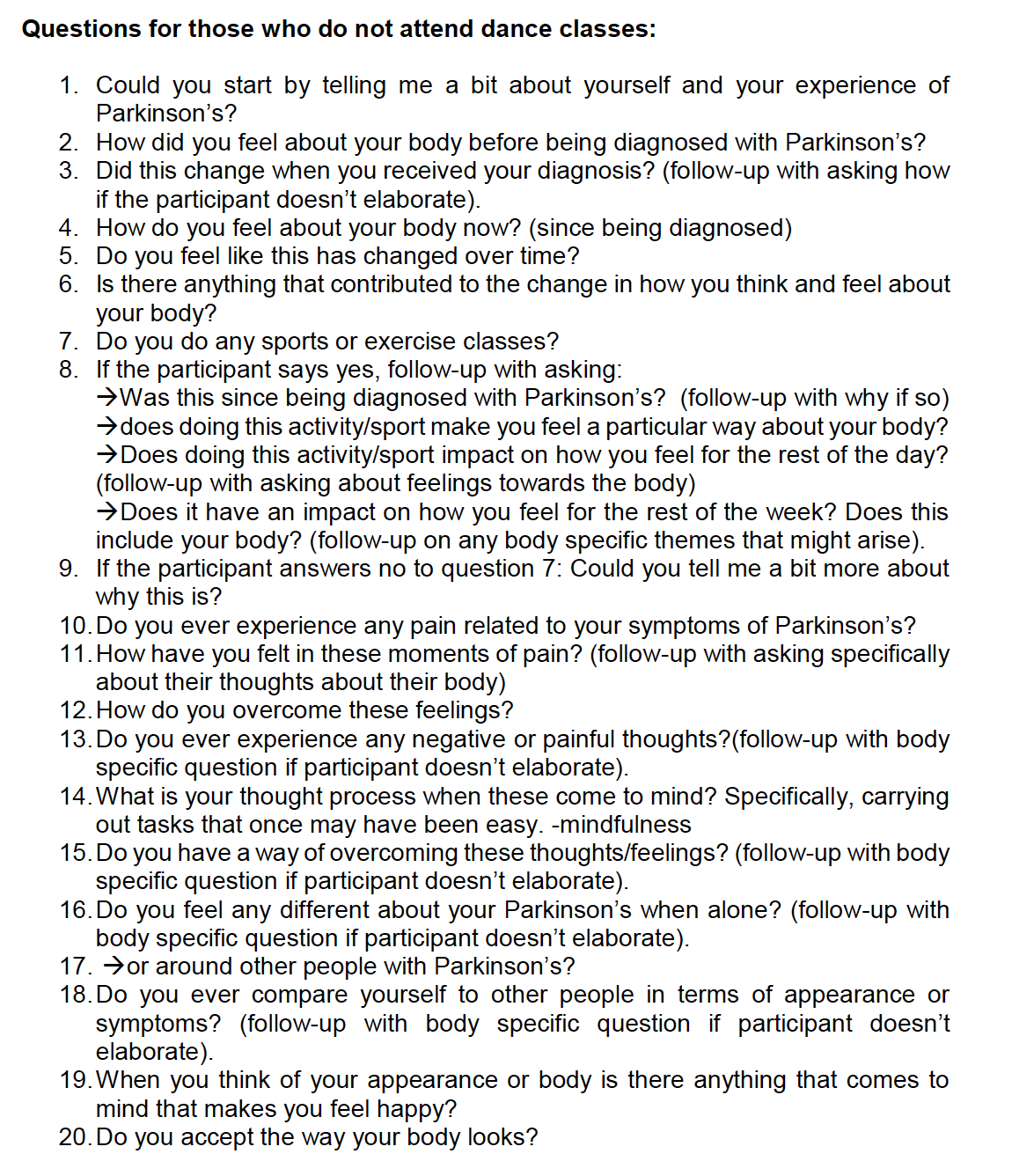


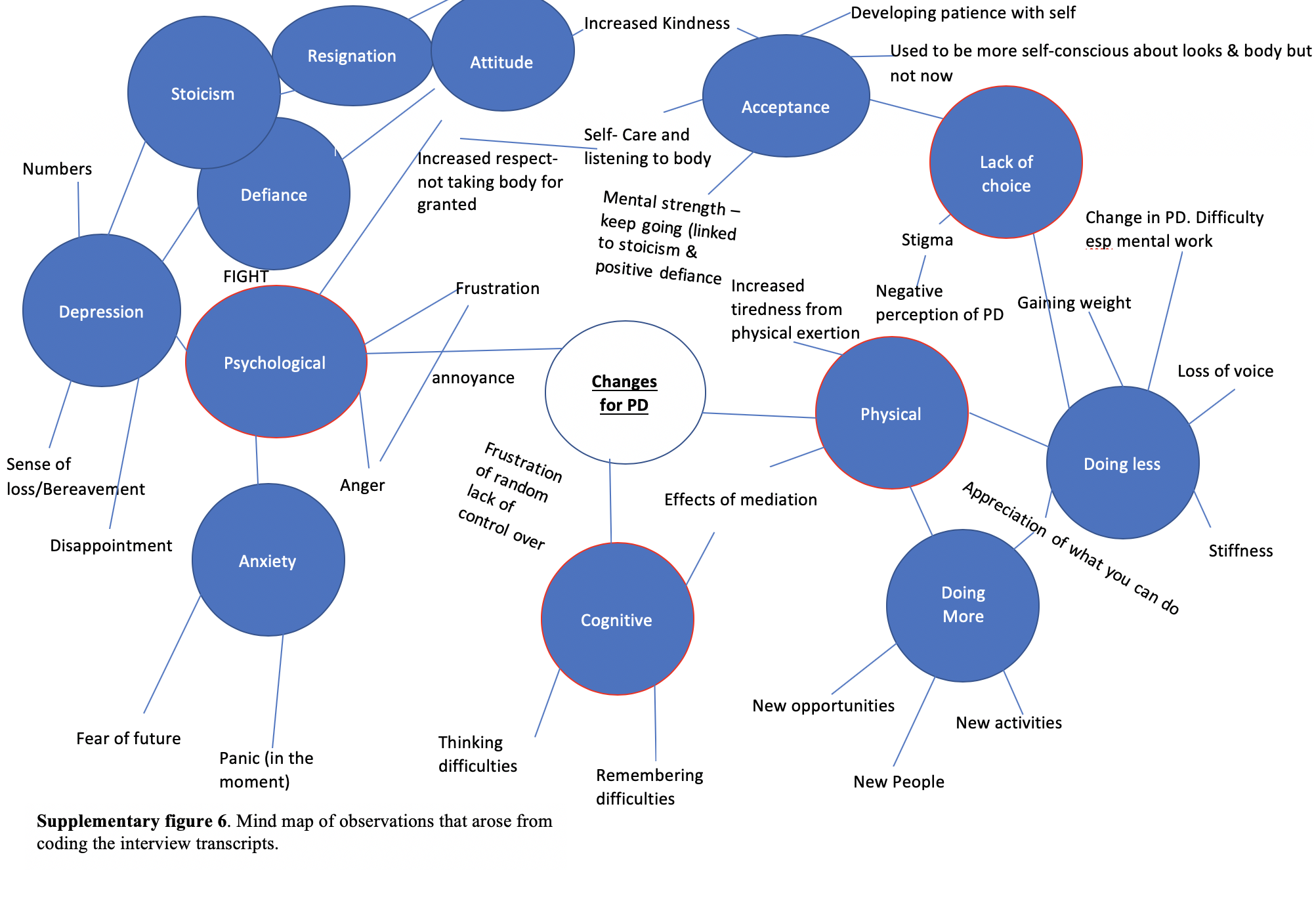


**Supplementary figure 6**. Mind map of observations that arose from coding the interview transcripts.

**Supplementary figure 7**. Mind map of observations that arose from coding the interview transcripts.

Interest in others’ lives

fun

It’s not exercise

Challenge to overcome

Seeing improvements

Harder to be positive when alone

Mixing with non-PD & making new friends

Encouraging

Seeing others makes you feel better

ABLENESS

(not disability)

Comparison to healthy

Comparison to PD

Seeing others inspiring

Lack of inhibition as all the same

If you don’t do anything=feel worse

STIMULATION

Motivating enjoyment

Help organise movements

DISTRACTION

Scheduling activities

Stretching

Understanding

Good tips

Isolation and turning away from PD

Overcoming loneliness
